# A meta-analysis of risk factors for a Dacron-cuffed catheter related infection in hemodialysis

**DOI:** 10.1186/s12882-024-03568-0

**Published:** 2024-04-08

**Authors:** Wen Chen, Zaoju Wang, Guoping Wang, Chunyu Cao, Bo Hong, Jinying Liu, Fuhua Xie, Runxiu Wang

**Affiliations:** 1 Department of Nephrology, The First Affiliated Hospital, Gannan Medical University, Ganzhou, 341000 Jiangxi China; 2 School of Basic Medicine, Gannan Medical University, Ganzhou, 341000 Jiangxi China

**Keywords:** Hemodialysis, Catheter-related infection, Risk factors, Meta-analysis

## Abstract

**Objective:**

To provide theoretical basis for prevention of a Dacron-cuffed catheter related infection (CRI), the risk factors of CRI in hemodialysis patients were systematically evaluated.

**Methods:**

Eight databases, including PubMed, Cochrane library, EMBASE, Web of Science, China National Knowledge Infrastructure (CNKI), Chinese Biomedical Database (CBM), Wanfang Database and Chinese Scientific Journal Database (VIP), were searched to screen out literatures related to the risk factors of long-term indwelling a Dacron-cuffed CRI in hemodialysis. Meta-analysis of risk factors for a Dacron-cuffed CRI in hemodialysis and publication bias test were performed using RevMan 5.4 software.

**Results:**

After screening, 13 literatures involving a Dacron-cuffed CRI were included, with a total of 625 patients, and the infection rate was 11.7%. The combined OR value and 95% confidence interval (CI) of all factors were: Combined with Diabetes (1.94, 1.51 ~ 2.50), Hb (1.82, 1.35 ~ 2.44), age (2.38, 1.06 ~ 5.34), catheter indwelling time (1.79, 1.21 ~ 2.66), serum albumin (2.26, 1.25 ~ 4.08), catheter indwelling site (3.29, 1.74 ~ 6.23) and the number of tube placement (5.40, 2.65 ~ 11.02).

**Conclusions:**

The main risk factors for a Dacron-cuffed CRI in hemodialysis were combined with diabetes, hemoglobin level, age, catheter indwelling time, serum albumin level, femoral vein catheter indwelling and catheterization times. In other words, hemodialysis patients are at higher risk of CRI if they have diabetes, or if they have a lower hemoglobin level, or if they are older, or if they have a longer duration of catheterization, or if they have a lower serum albumin level, or if they have a femoral vein catheter, or if they have more catheters.

## Background

Long-term catheters for hemodialysis are also known as tunneled central venous catheters (TCVC). Dacron sleeves are installed outside the catheters. When the dacron sleeves are inserted, a subcutaneous tunnel needs to be made. It can also reduce the catheter with dacron sleeve protruding. TCVC catheterization is widely used in clinic because it solves the problem of poor vascular condition and no vascular channel due to repeated fistula failures. However, due to the long catheter indwelling time, the catheter-related infection (CRI) rate increases, which adversely affects the prognosis and also increases the risk of death in hemodialysis patients [1]. Here, we used a meta-analysis to explore the risk factors associated with TCVC CRI in hemodialysis to provide theoretical basis for prevention and treatment of CRI.

## Materials and methods

### Search strategy

A computer-assisted study search of 8 databases, including PubMed, Cochrane library, EMBASE, Web of Science, CNKI, CBM, Wanfang Database and VIP was performed to search for articles in any language, with the time range of retrieval set between the establishment of the database to February 28, 2021. We used the following medical search heading (MeSH) terms and search strings: (Catheter Related Infections OR Catheter-Related Infection OR Infection, Catheter-Related OR Infections, Catheter- Related OR Catheter-Associated Infections OR Catheter Associated Infections OR Catheter-Associated Infection OR Infection, Catheter-Associated OR Infections, Catheter-Associated) AND (Dialyses, Renal OR Renal Dialyses OR Dialysis, Renal OR Hemodialysis OR Hemodialyses OR Dialysis, Extracorporeal OR Dialyses, Extracorporeal OR Extracorporeal Dialyses OR Extracorporeal Dialysis).

### Inclusion criteria

(1) Published studies on CRI and its risk factors in hemodialysis at home and abroad, among which the types of CRI included bacteremia, local infection at the exit site and tunnel, and systemic infection. (2)The subjects were hemodialysis patients with TCVC catheterization, and the types of catheterization were not limited. (3)The study method was case–control study or cohort study. (4)The research hypotheses of various literatures are similar. (5) Multivariate logistic regression analysis was used to provide OR value and 95%CI OR could be converted to OR value and 95%CI.

### Exclusion criteria

(1) Case reports or reviews or animal experiments. (2) Duplicate published studies. (3) The type of catheter placement was not specified. (4) Data could not be extracted. (5) The outcome indicators were inconsistent.

### Literature screening and data extraction

Two researchers independently screened the literature according to the inclusion and exclusion criteria, and extracted the first author, region, publication time, study method, catheter indwelling time, number of cases, number of controls, risk factors, OR value, 95% CI and other information.

### Quality assessment

Newcastle–Ottawa Scale (NOS), an observational quality assessment tool, was used to evaluate literature quality. The evaluation indicators included 8 items: whether the cases were included properly, whether the cases were representative, control selection, control determination, comparability between groups, exposure factors, whether the assessment methods of exposure were the same between groups, and non-response rate. Out of 9 stars, ≥ 6 stars indicated that the literature quality is generally high. The two researchers made independent evaluation and discussed. In case of disagreement, they discussed with the third researcher to make a decision. Two researchers independently evaluated and discussed the above indicators. In case of disagreement, they would discuss and decide with the third researcher.

### Statistical analysis

RevMan5.4 software was used for meta-analysis and publication bias test. The combined OR value and 95% CI of risk factors were calculated. The heterogeneity was expressed by I^2^ value. If there was no heterogeneity in the study (*P* > 0.1, I^2^ < 50%), the fixed-effect model was used to combine the effect size. If heterogeneity existed, the random effects model was used to combine the effect size. Sensitivity analysis was used to detect the stability of the results. Funnel plot was used to test the publication bias of the literature. *P* < 0.05 was considered statistically significant.

## Results

### Characteristics, quality and combined meta-analysis

**Fig. 1:**
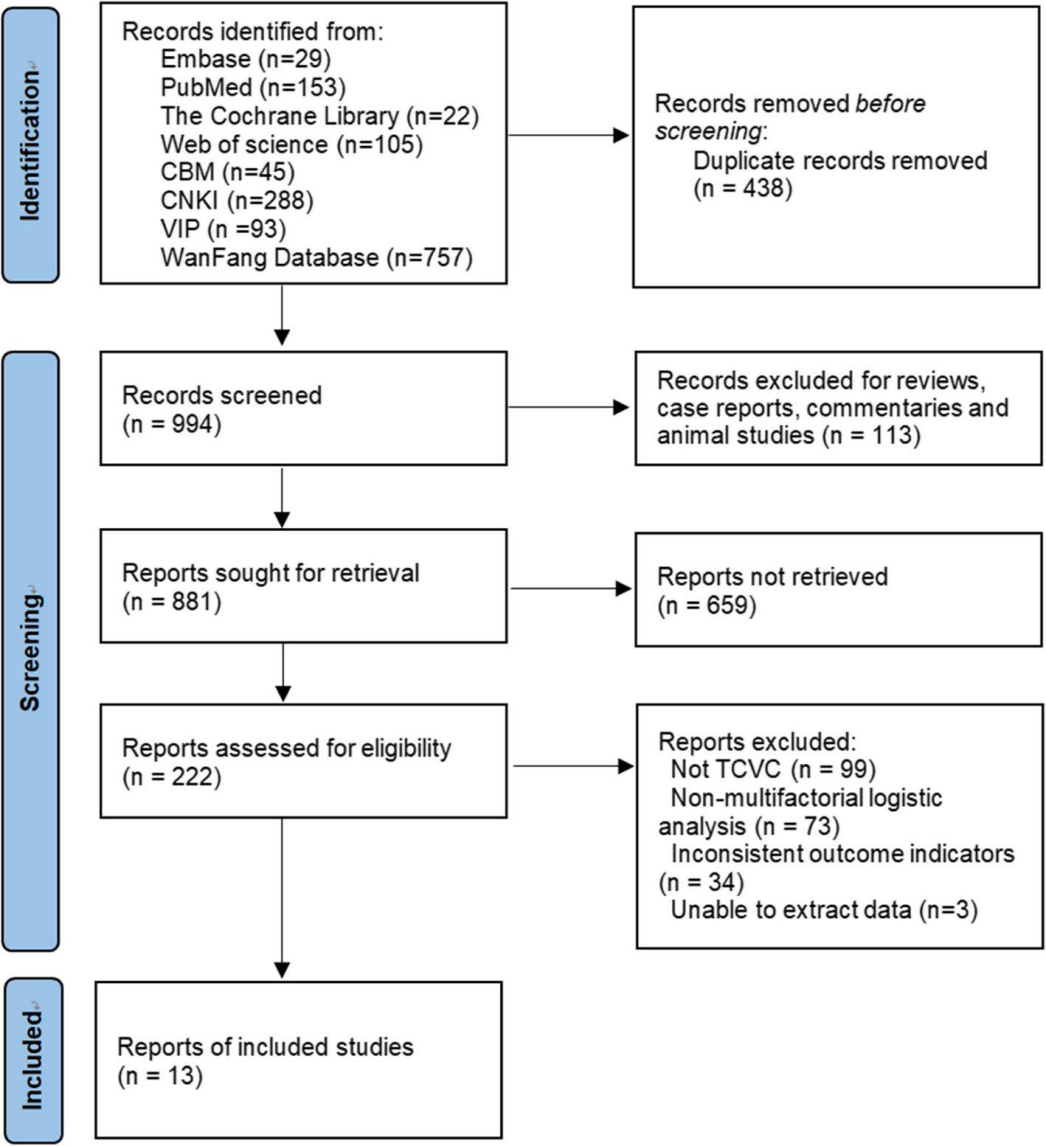
Literatures screening process and results

Evaluation of the Included Studies A total of 1432 literatures were retrieved and screened according to inclusion and exclusion criteria. Finally, 13 literatures were included [2–14], of which 10 were in Chinese and 3 were in English (as shown in Fig. 1). All the included literatures were case–control studies, with a total of 5339 subjects, 625 of whom developed hemodialysis CRI (the infection rate was 11.7%). The basic characteristics of the included studies were shown in Table 1, and the quality evaluation of the methods was shown in Table 2. Combined Meta-analysis showed that there were seven risk factors for catheter-associated infections in hemodialysis (*P* < 0.05), as shown in Table 3.

**Table 1:** Basic features of the included literature

**Reference**	**Age (years)**	**Catheter indwelling time** **Time, number**		**Microorganism causing the bacteremia**		
				**G**^**+**^	**G**^**−**^	**Fungus**
				**n(%)**	**n(%)**	**n(%)**
Martin et al. (Ref [2])	58 (median)	≤ 90 d, 18; > 90 d, 21		23 (59%)	10 (26%)	3 (8%)
Wang et al. (Ref [3])	55.2 ± 10.9	≤ 7 d, 2; 8–14 d, 9; ≥ 15 d, 71		Not mention		
Cao et al. (Ref [4])	< 60 y, 41 cases ≥ 60 y, 37 cases	< 6 m, 16; 6–12 m, 23; ≥ 12 m, 39		Not mention		
Hua et al. (Ref [5])	70.1 ± 8.4	Not mention		19 (61.29%)	12 (38.71%)	0 (0%)
Tian et al. (Ref [6])	71.8 ± 13.4	< 12 m, 12; 12–24 m, 34; ≥ 24 m, 21		60 (63.80%)	28 (29.80%)	6 (6.40%)
Sun et al. (Ref [7])	56.7 ± 11.5	≤ 7 d, 1; 8–14 d, 4; ≥ 15 d, 30		Not mention		
Li et al. (Ref [8])	< 60 y, 24 cases ≥ 60 y, 11 cases	< 5 w, 20; ≥ 5 w, 15		51 (28%)	48 (72%)	0 (0%)
Yang et al. (Ref [9])	71.2 ± 4.3	≥ 3 w		17 (80.95%)	4 (19.05%)	0 (0%)
Zhao et al. (Ref [10])	< 55 y, 51 cases ≥ 55 y, 10 cases	< 36 m, 35; ≥ 36 m, 26		40 (59.70%)	25 (37.30%)	2 (2.99%)
Huang et al. (Ref [11])	79.3 ± 1.9	4.9 ± 4.7 m, 7		6 (85.71%)	1 (14.29%)	0 (0%)
Izoard et al. (Ref [12])	69 ± 14	> 10 m		768 (73%)	42 (4%)	0 (0%)
Di et al. (Ref [13])	67.5 ± 14.2	> 90 d		Not mention		
Lemaire et al. (Ref [14])	< 72 y, 14 cases ≥ 72 y, 48 cases	> 90 d		171 (76%)	35 (15%)	20 (9%)
**Reference**	**Year**	**Region**	**Type of Research**	**Cases**	**Controls**	**Risk factors**
Martin et al. (Ref [2])	2020	Australia	Case control	39	188	a
Wang et al. (Ref [3])	2019	China	Case control	82	368	a, b, c, d
Cao et al. (Ref [4])	2019	China	Case control	78	236	a, e
Hua et al. (Ref [5])	2019	China	Case control	31	125	b, c, e, f
Tian et al. (Ref [6])	2018	China	Case control	30	130	a, b, c, d, e
Sun et al. (Ref [7])	2018	China	Case control	35	180	a, b, c, d
Li et al. (Ref [8])	2018	China	Case control	35	45	a, b, d, e, f, g
Yang et al. (Ref [9])	2018	China	Case control	21	168	a, c
Zhao et al. (Ref [10])	2017	China	Case control	61	85	a, b
Huang et al. (Ref [11])	2017	China	Case control	7	71	b, g
Izoard et al. (Ref [12])	2017	France	Case control	102	812	a
Di et al. (Ref [13])	2014	China	Case control	42	138	a, b, d, e
Lemaire et al. (Ref [14])	2009	France	Case control	62	2168	a, d

*Notes*: *Ref* Reference; a: combined with diabetes; b: Hb; c: age; d: duration of catheterization; e: serum albumin; f: catheterization site; g: number of catheterizations. y: years; d: days; w: weeks; m: months; G^+^: positive gram; G^−^: negative gram

**Table 2:** Quality evaluation of Newcastle–Ottawa Scale

Reference	Selection	Comparability	Outcome	Total Score
Martin et al. (Ref [2])	★★	★★	★★★	7
Wang et al. (Ref [3])	★★	★★	★★★	7
Cao et al. (Ref [4])	★★	★★	★★	6
Hua et al. (Ref [5])	★★	★★	★★★	7
Tian et al. (Ref [6])	★★	★★	★★	6
Sun et al. (Ref [7])	★★	★★	★★★	7
Li et al. (Ref [8])	★★	★★	★★★	7
Yang et al. (Ref [9])	★★	★★	★★★	6
Zhao et al. (Ref [10])	★★	★★	★★★	7
Huang et al. (Ref [11])	★★	★★	★★	6
Izoard et al. (Ref [12])	★★★	★★	★★★	8
Di et al. (Ref [13])	★★	★★	★★	6
Lemaire et al. (Ref [14])	★★	★★	★★★	7

**Table 3:** Meta-analysis results of the combination of risk factors for CRI

**Risk factors**	**Number of studies**	**Heterogeneity test**		**Model**	**OR**	**95% CI**	***p*-value**
		**I**^**2**^**(%)**	***p*-value**				
Combined with diabetes	11	61	0.00500	Random	1.96	1.56 ~ 2.48	< 0.00001
Hb level	8	91	< 0.00001	Random	1.82	1.35 ~ 2.45	< 0.00010
Age	5	96	< 0.00001	Random	2.38	1.06 ~ 5.34	0.04000
Catheter indwelling time	6	86	< 0.00001	Random	1.79	1.21 ~ 2.66	0.00400
Serum albumin level	5	90	< 0.00001	Random	2.26	1.25 ~ 4.08	0.00700
Femoral vein catheter	2	0	0.88000	Fixed	3.29	1.74 ~ 6.23	0.00030
Catheterization times	2	27	0.24000	Fixed	5.40	2.65 ~ 11.02	< 0.00001

### Combined with diabetes and CRI

**Fig. 2:**
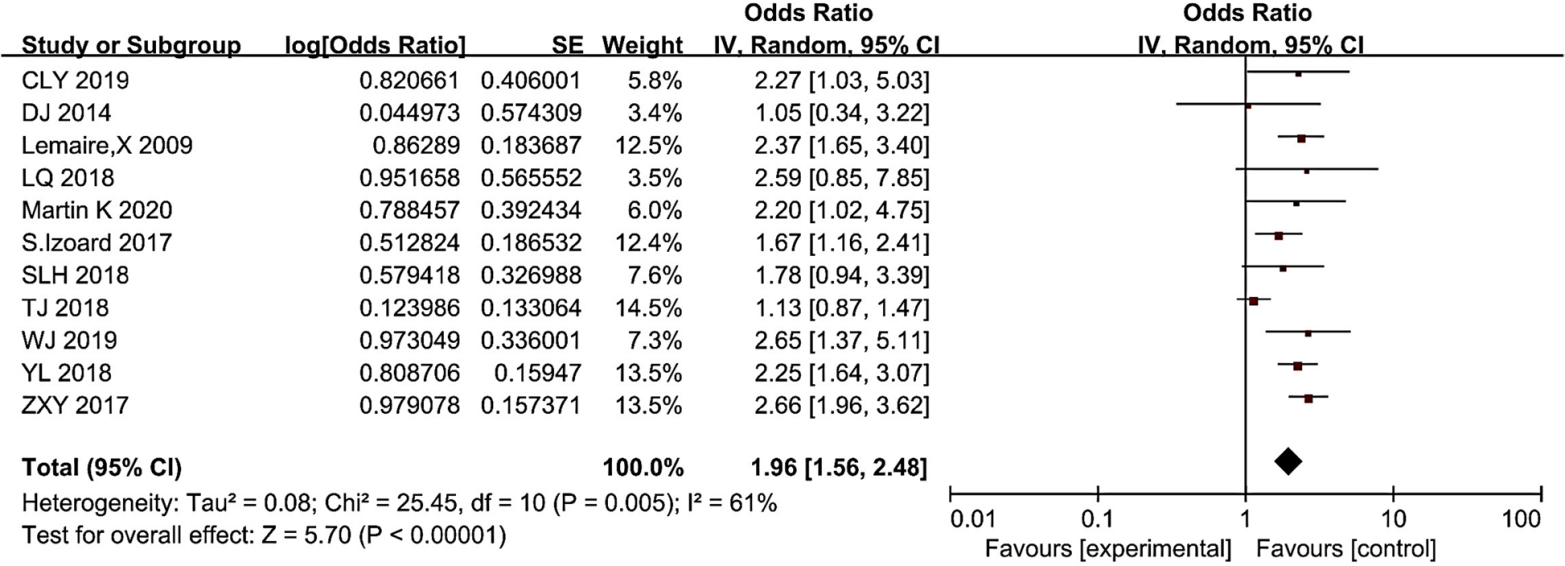
Meta-analysis of combined with diabetes in relation to the occurrence of CRI. The figure showed that the risk of CRI in diabetic patients was 1.94 (1.51 ~ 2.50) times higher than in non-diabetic patients, *p*-value < 0.00001

Seen from Fig. 2, the results of the study were heterogeneous, with I^2^ = 58% (> 50%), but within the acceptable range. Therefore, the random effects model was selected for meta-analysis with diabetes factors, and the results showed that diabetes was a risk factor for CRI in hemodialysis patients (*P* < 0.05). Overall, the risk of CRI in diabetic patients was 1.94 (1.51 ~ 2.50) times than that of non-diabetic patients.

### Hb and CRI

**Fig. 3:**
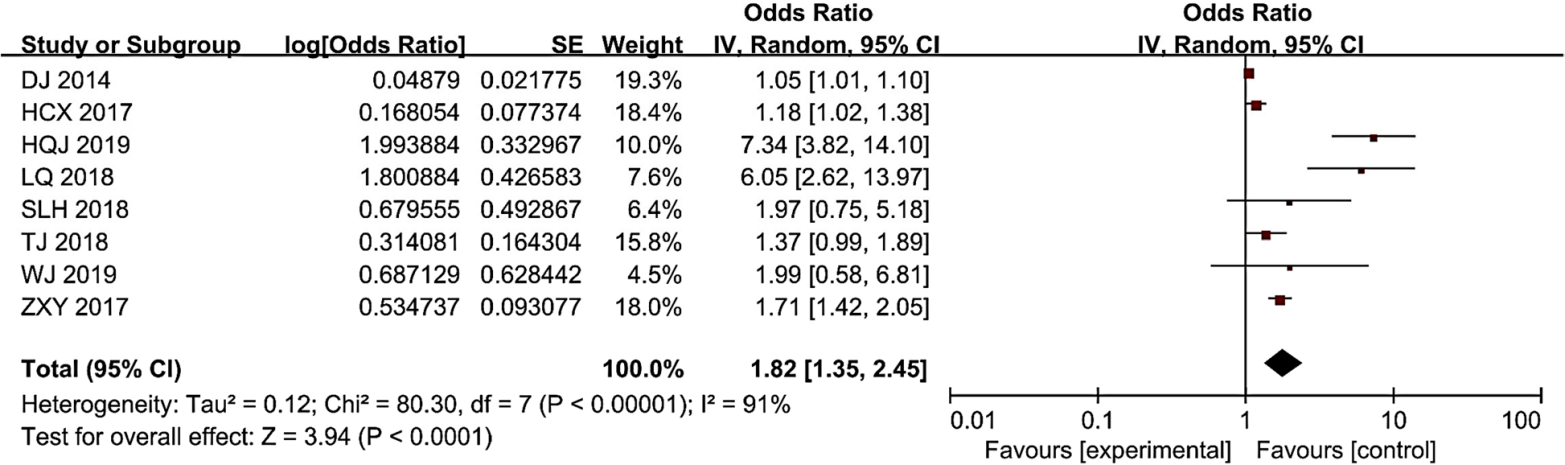
Meta-analysis of Hb in relation to the occurrence of CRI

As can be seen from Fig. 3, there was heterogeneity in the study results, with I^2^ = 91% (> 50%). Therefore, the random effects model was selected for the meta-analysis of Hb factor, indicating that Hb is a risk factor for CRI in hemodialysis patients (*P* < 0.05). The risk of CRI in patients with low hemoglobin was 1.82 (1.35 ~ 2.44) times than that of patients with non-low hemoglobin.

### Age and CRI

**Fig. 4:**
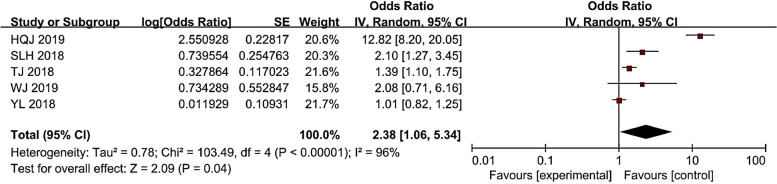
Meta-analysis of age in relation to the occurrence of CRI

As shown in Fig. 4, the results of the study were heterogeneous, with I^2^ = 96% (> 50%). Therefore, the random effects model was used to conduct a meta-analysis of age factors, and it was finally concluded that age was a risk factor for CRI in hemodialysis patients (*P* < 0.05). Overall, the risk of CRI in older patients was 2.38 (1.06 ~ 5.34) times than that of younger patients.

### Catheter indwelling time and CRI

**Fig. 5:**
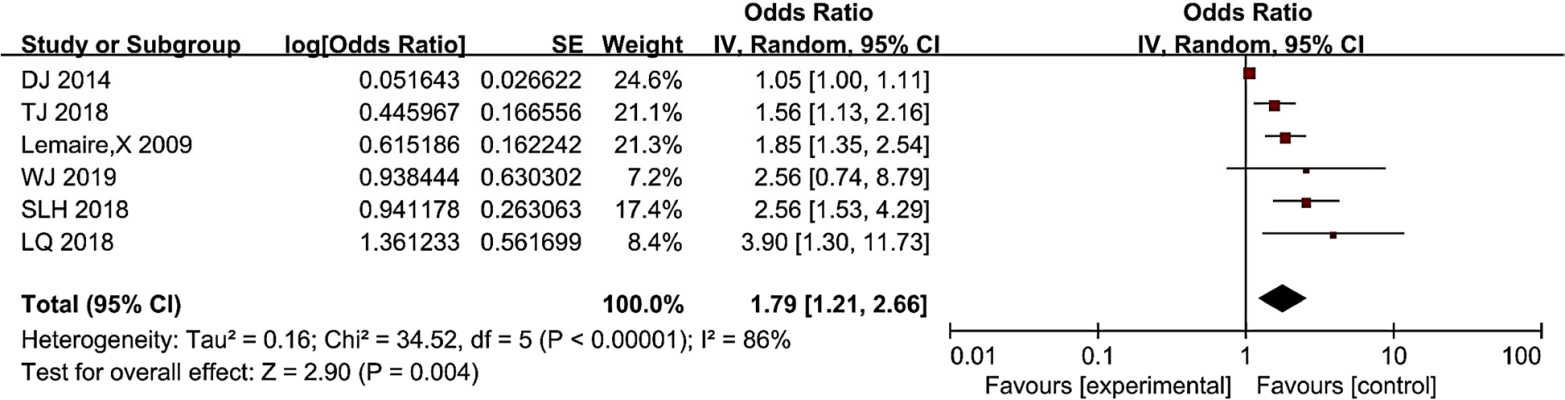
Meta-analysis of catheter indwelling time in relation to the occurrence of CRI

As shown in Fig. 5, there was heterogeneity in the study results, with I^2^ = 86% (> 50%). Therefore, the random-effects model was selected for the meta-analysis of catheter indwelling time, indicating that catheter indwelling time was a risk factor for CRI in hemodialysis patients (*P* < 0.05). Overall, the risk of catheter-associated infection was 1.79 (1.21 ~ 2.66) times higher in patients with long catheter indwelling than in patients with short catheter indwelling.

### Serum albumin and CRI

**Fig. 6:**
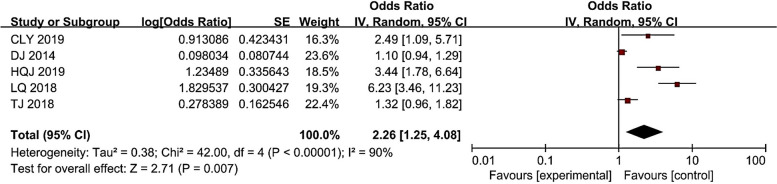
Meta-analysis of serum albumin in relation to the occurrence of CRI

As shown in Fig. 6, there was heterogeneity in the study results, with I^2^ = 90% (> 50%). Therefore, the random effects model was selected for serum albumin factor meta-analysis, and the results showed that low serum albumin was a risk factor for catheter-associated infection in hemodialysis patients (*P* < 0.05). Overall, patients with hypoproteinemia had 2.26 (1.25 ~ 4.08) times the risk of CRI compared with patients with normal serum albumin.

### Catheter indwelling site and CRI

**Fig. 7:**
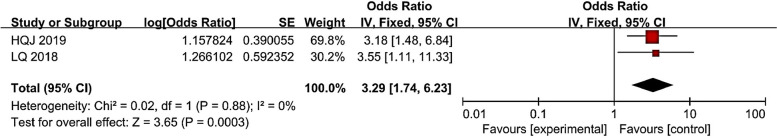
Meta-analysis of indwelling site of catheter in relation to the occurrence of CRI

As shown in Fig. 7, there was heterogeneity in the study results, with I^2^ = 0% (< 50%). Therefore, the fixed effect model was selected for meta-analysis of catheter indwelling site factors. The results showed that catheter indwelling site was a risk factor for CRI in hemodialysis patients (*P* < 0.05). Overall, the risk of CRI in patients with femoral vein catheterization was 3.29 (1.74 ~ 6.23) times higher than that in patients with other catheter site.

### Number of catheterization and CRI

**Fig. 8:**
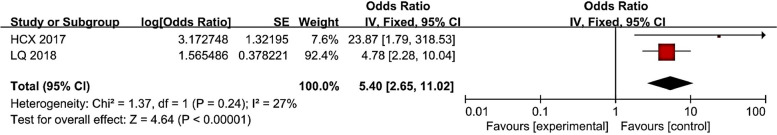
Meta-analysis of the number of tube in relation to the occurrence of CRI

As shown in Fig. 8, it can be clearly seen that I^2^ = 27% (< 50%). Therefore, the fixed effect model was selected for meta-analysis of the number of catheterization factors, and the results showed that the number of catheterization was a risk factor for catheter-related infection in hemodialysis patients (*P* < 0.05). Overall, the risk of CRI in patients with more catheterization was 5.40 (2.65 ~ 11.02) times than that of patients with less catheterization.

### Sensitivity analysis

Fixed effects model and random effects model were used to estimate the combined OR value and 95% CI for risk factors of CRI. The results are shown in Table 4, and the values are very close, which indicates that the combined results of this study are basically reliable.

**Table 4:** Sensitivity analysis results

Risk factors	OR_fixed_(95% CI)	OR_random_(95%CI)
Combined with diabetes	1.88 (1.65 ~ 2.14)	1.96 (1.56 ~ 2.48)
Hb level	1.10 (1.06 ~ 1.15)	1.82 (1.35 ~ 2.45)
Age	1.57 (1.36 ~ 1.81)	2.38 (1.06 ~ 5.34)
Catheter indwelling time	1.09 (1.04 ~ 1.15)	1.79 (1.21 ~ 2.66)
Serum albumin level	1.33 (1.16 ~ 1.51)	2.26 (1.25 ~ 4.08)
Femoral vein catheter	3.29 (1.74 ~ 6.23)	3.29 (1.74 ~ 6.23)
Catheterization times	5.40 (2.65 ~ 11.02)	6.49 (1.89 ~ 22.30)

### Publication bias analysis

**Fig. 9:**
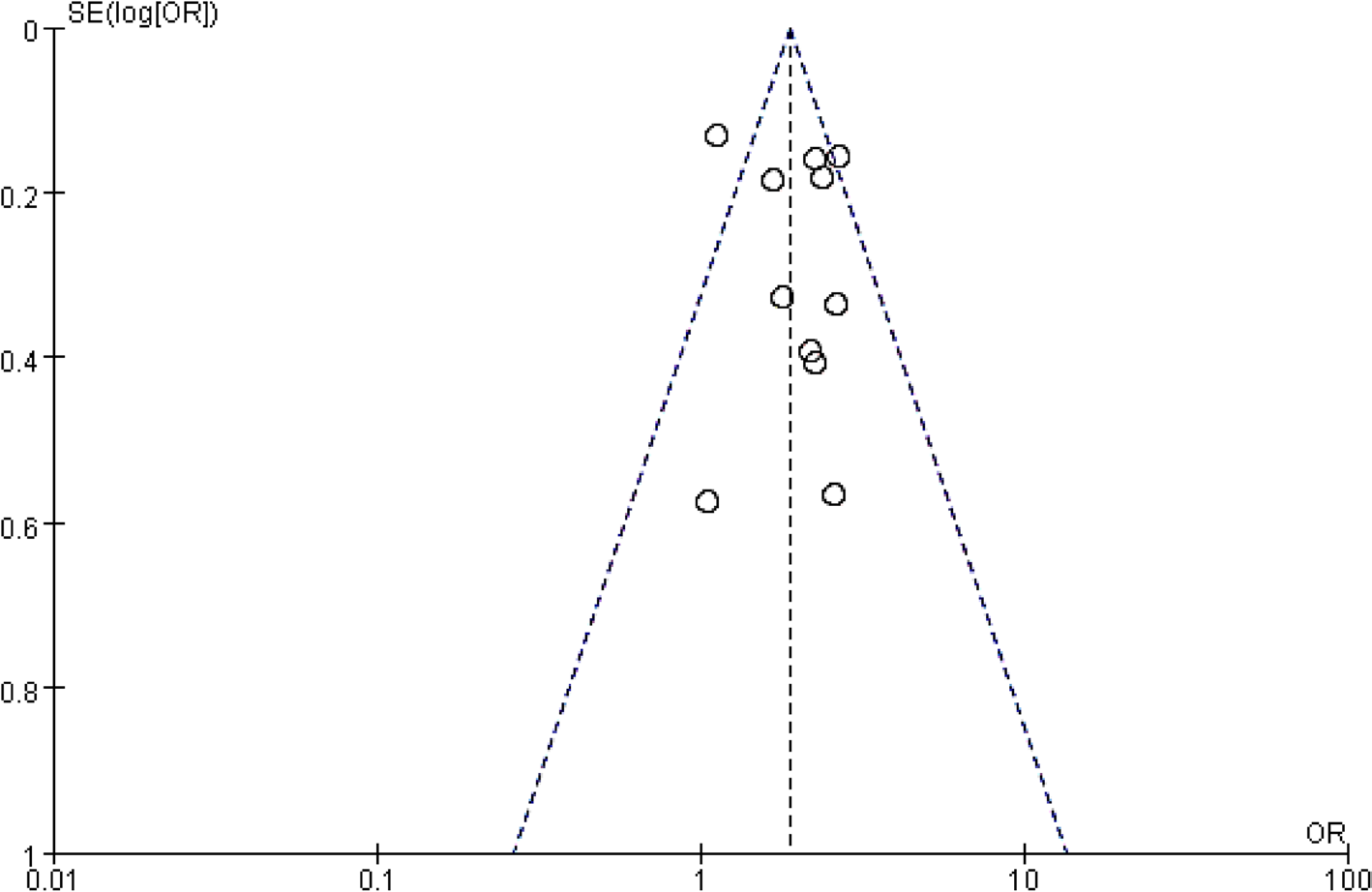
Funnel plot for diabetes analysis

Publication bias analysis was performed on literatures corresponding to each research factor one by one, and it was found that funnel plots of risk factors of catheter-associated infection were basically symmetric, indicating that the results of meta-analysis were stable. Taking the results of the funnel plot of risk factors combined with diabetes as an example, the included 11 literatures were basically within 95% CI and were distributed symmetrically in an inverted funnel shape, suggesting no obvious publication bias (Fig. 9).

## Discussion

Although TCVC overcomes some shortcomings of deep venous catheter and arteriovenous fistula, it also increases the risk of CRI due to long-term indwelling catheter. Infection is one of the known and serious complications of hemodialysis, which can easily induce other complications and lead to the death of patients [15]. Therefore, it is extremely important to correctly understand the risk factors of CRI and actively prevent them. We used meta-analysis to find that the main risk factors of CRI in hemodialysis are combined-diabetes, age, hemoglobin level, catheter indwelling time, serum albumin level, femoral vein catheterization and catheterization times.

Studies [16] have shown that patients with a central venous catheter have a higher incidence of infectious complications than those with a fistula. The results of our analysis showed that the number of catheterization was one of the main risk factors for the occurrence of CRI, and the OR value reached 5.40 (2.65 ~ 11.02). Repeated puncture during catheterization could cause damage to subcutaneous tissues and the inner wall of blood vessels, thus increasing the opportunity for bacterial invasion [17]. Among patients receiving continuous hemodialysis, the incidence of CRI is relatively low, and it is not significantly affected by the first new vein puncture [18]. Therefore, it is necessary to strengthen the operation proficiency of catheterization personnel, strive to use the least number of catheterization times, and achieve satisfactory catheterization effect. The more skilled the catheterist is, the fewer vascular insertions will be made, and the more strictly regulated aseptic procedures will reduce infection [19]. In addition, due to the lack of long-term indwelling catheter nursing awareness and professional knowledge, non-inpatient patients with TCVC catheterization have poor awareness of catheter maintenance, which will increase the risk of infection when the catheter outlet site is flooded, the film is damaged and loose, etc., such as unplanned operations such as extubation and re-catheterization.

Our study found that the OR values of CRI at the site of catheter indwelling (femoral vein catheterization) and diabetes were 3.29 and 1.94, respectively, second only to the number of catheterization. Patients with this kind of disease should develop good personal hygiene habits, keep the catheterization site dry and clean, and patients with femoral vein catheterization can easily lead to indwelling catheter slip due to vigorous activities when getting up. Therefore, all patients, especially those discharged from hospital with catheter, should be provided with standard education on infection prevention, and patients with femoral vein catheterization should be advised to exercise as little as possible and pay attention to personal hygiene.

Our study showed that long catheter indwelling time was an important independent risk factor for CRI. Some studies [20] have shown that patients undergoing hemodialysis through tunnel catheterization are prone to Gram-positive CRI, and the pathogenic bacteria are mainly coagulase negative Staphylococcus, and the pathogens mainly come from the skin of the puncture site and the hands of catheterizers. During summer catheterization, the reproduction rate of pathogenic microorganisms in the operating environment can be significantly accelerated due to the high ambient temperature, while during winter catheterization, the incidence rate of CRI can be increased due to the reduced frequency of skin cleaning. Therefore, sealing the tube with citrate combined with antibiotics during catheterization can reduce the risk of infection associated with long-term indwelling catheter [21]. When catheter infection is highly suspected, intravenous empiric antibiotic treatment is given immediately and antibiotic type is adjusted according to blood culture results. For clinical staff, hand hygiene should be checked monthly and hand hygiene reports should be shared. Other studies [22] confirmed that there was a positive correlation between the duration of catheter indentation and the occurrence of CRI, with the duration of catheter indentation > 15d, and the incidence of CRI could reach more than 20%. The catheter can not only form a channel connecting the body with the outside world, which can provide convenience for pathogen invasion, but also, due to the repeated opening of the port, pathogens can successfully invade the blood circulation and cause systemic infection through the blood circulation [23].

We found that low serum albumin was another important risk factor for CRI, and the OR value of low serum albumin for CRI was 2.26. Albumin can effectively evaluate the nutritional status and death risk of dialysis patients, and patients with low albumin have a higher risk of infection [24]. In our study, age and low hemoglobin level were also independent risk factors for CRI, with OR values of 2.38 and 1.82, respectively, which reflected the patient's physical condition and nutritional level. As people age, they are more likely to experience multiple organ dysfunction, malnutrition, weakened and weakened immune function, which increases the risk of catheter infection. More than 500,000 Chinese patients have received renal replacement therapy, and more than 90% of them suffer from renal anemia. Anemia can increase the incidence of cardiovascular complications in dialysis patients, reduce the quality of life and increase the mortality of dialysis patients. Moreover, anemia reflects the poor nutritional status of patients, weak resistance and easy occurrence of CRI. Therefore, in clinical work, it is necessary to improve the relevant examination of pre-dialysis albumin and hemoglobin, arouse the attention of patients and their families, and timely improve the malnutrition of pre-dialysis patients, so as to reduce the infection rate and mortality of hemodialysis patients.

In clinical work, it is found that CRI is not only affected by a single factor. TCVC hemodialysis patients are mostly patients with advanced renal disease complicated with other underlying diseases, and the interaction between multiple risk factors often leads to increased difficulties in clinical treatment. In addition to the 7 risk factors identified in this study, another important factor in the occurrence of catheter-associated infection in TCVC is whether stereosis is strictly observed during the use of TCVC and whether the TCVC tunnel is properly protected. Studies have shown that the initiation of standardized nursing protocols and the regular review of compliance with the protocols can significantly reduce the CRI rate of hemodialysis patients [25]. Therefore, education and ability inspection of medical staff in dialysis room should be strengthened. Catheterization personnel should strictly grasp indications of tunnel catheterization and standardize catheterization operation. Nursing staff should carry out strict nursing process to reduce infection caused by improper operation. In addition, education and participation of patients and their families should be strengthened. Joint efforts between health care providers and patients are essential to prevent CRI. For routine infection prevention, an infection control team, namely an infection monitoring team, can be established to monitor and track CRI (electronic database) and help prevent and evaluate the consequences of pathway infection [26].

At present, there are few Meta-analyses on CRI in hemodialysis at home and abroad. Our study had strict inclusion and exclusion criteria. We selected patients with long-term central vein catheterization, namely TCVC catheterization, and selected literatures with logistic multivariate regression analysis, which reduced the influence of confounding factors to a certain extent. The results of our analysis provide a basis for clinical prevention and treatment of CRI in hemodialysis. However, due to the influence of the population and case selection, the results of this meta-analysis need to be verified by multi-center and prospective studies. Moreover, due to inconsistent data recording methods in the included literature, the incidence and risk factors of CRI with different TCVC indwelling times could not be analyzed, and also the lack of consensus on the definition of the risk factors studied and that this heterogeneity influences the generalization of the results derived from the review.

## Conclusions

In summary, our present evidence shows that the main risk factors of CRI in hemodialysis were combined with diabetes, hemoglobin level, age, catheter indwelling time, serum albumin level, femoral vein catheter indwelling and catheterization times. In other words, hemodialysis patients are at higher risk of CRI if they have diabetes, or if they have a lower hemoglobin level, or if they are older, or if they have a longer duration of catheterization, or if they have a lower serum albumin level, or if they have a femoral vein catheter, or if they have more catheters.

## Data Availability

The datasets used and analysed during the current study available from the corresponding author on reasonable request.
